# Prevalence and Outcomes of Online Sexual Activities Among Women and Men in Germany: A National Online Survey

**DOI:** 10.1007/s10508-025-03353-6

**Published:** 2025-12-27

**Authors:** Nicola Döring, Veronika Mikhailova, Denise Schönnenbeck

**Affiliations:** Department of Economic Sciences and Media, Technische Universität Ilmenau, Ehrenbergstr. 29, 98693 Ilmenau, Germany

**Keywords:** Online sexual activities, Gender, Sexual behavior, Sexual health, Online sexuality

## Abstract

Online sexual activities (OSA) are widespread and linked to both benefits and risks for sexual health. Experiences with OSA often differ by gender, potentially leading to distinct perceived outcomes. Understanding these differences is essential for tailoring education, prevention, and intervention strategies. However, current data on OSA use and their subjective outcomes among adults in Germany are limited. To address this gap, this study aimed to assess gender-specific prevalence, experiences, and perceived outcomes of OSA. A national online sample of 2832 adults in Germany (aged 18–65; 50% women, 50% men) reported in December 2022 on their 12-month prevalence of OSA overall, their engagement and subjective experiences with specific types of OSA, and the overall perception of positive and negative impact of OSA on their personal lives. A total of 62% of participants reported engaging in at least one type of OSA in the past 12 months, with sexual entertainment and sexual information being the most common types. Men were significantly more likely than women to engage in OSA overall and across most types, except for purchasing sex-related products. Subjective experiences with specific types of OSA were predominantly positive and largely similar between genders. Both men and women generally evaluated the impact of OSA on their personal lives as more positive than negative, with men reporting slightly higher perceived benefits. Results illustrate that OSA are common and generally experienced positively among men and women. Sexual health professionals need to address not only potential risks but also the personal benefits of OSA in their work.

## Introduction

Since the mid-1990s, when the Internet became widely available to the general population, online sexual activities (OSA) have become increasingly popular, reflecting nearly three decades of integration into everyday sexual life (Ballester-Arnal et al., 2021; Kim & Berdychevsky, 2025). OSA include practices such as accessing online pornography, using dating platforms or apps, and exchanging nude selfies with sexual partners (sexting; Döring & Mohseni, 2018). While OSA remains the most prevalent umbrella term in academic literature, other labels are also used, including Internet sexuality, sexuality on the Internet, online sexuality, mobile sexuality, cybersexuality, digital sexuality, digisexualities, and sexuality in digital media (Desbuleux et al., 2025; Döring, 2009, 2012; Griffin-Shelley, 2003; McArthur & Twist, 2017).

There is no overarching theory that comprehensively describes and explains OSA. Instead, domain-specific theoretical approaches must be adapted and extended for the respective sexual activity (e.g., theories of pornography use, theories of sexual partner seeking, theories of intimate interpersonal communication) and the specific digital context in which it occurs (e.g., social media platforms, smartphone apps, virtual reality environments; Döring et al., 2021).

In both public and academic discourse, two dominant theoretical perspectives can be identified when it comes to evaluating experiences and outcomes of OSA:

A benefit-oriented perspective regards the digitalization of sexuality as a relatively normal and largely benign or even beneficial extension of sexual behavior in the general population. This perspective is evident in studies that explicitly consider the benefits of OSA and systematically argue against a negativity bias (Döring, 2009; Kim & Berdychevsky, 2025; Naezer, 2018). The benefit-oriented perspective calls for a more sex-positive (Williams et al., 2015) and technology-positive (Riva et al., 2012) OSA approach—one that does not ignore potential harms but considers risks and opportunities in a balanced way (Döring & Mohseni, 2018). Some researchers even argue that OSA holds empowerment potential for women’s sexual expression (Döring, 2000).

In contrast, a risk-oriented perspective tends to frame the shift of sexual activities into digital contexts as mainly risky behavior, thereby focusing on online sexual problems. This view emphasizes potential harms such as addiction, sexualized violence, exploitation, or infidelity (Cooper et al., 2004; Hertlein & Cravens, 2014; Lunde & Joleby, 2021; Machimbarrena et al., 2018). It is reflected in the large number of studies and measurement instruments (Eleuteri et al., 2014) that primarily focus on problematic or pathological aspects of OSA (e.g., compulsive online pornography use scales, online sexual victimization measures). Gendered risks are often highlighted, particularly the sexual victimization of women in the context of OSA (Döring, 2000).

Gender may shape access to both benefits and risks of OSA. For example, men are more often portrayed as active seekers of sexual stimulation, while women’s experiences may be more relational, contextual, and shaped by sociocultural norms (Kim & Berdychevsky, 2025). These asymmetries suggest that perceived outcomes of OSA may differ by gender, both in terms of exposure to risks and in the type and framing of positive experiences (Döring et al., 2021).

Against this background, the current study investigates how common OSA are among male and female Internet users in Germany and how individuals perceive their engagement in them. While previous studies have examined OSA and their perceived outcomes in samples from Germany (e.g., Döring & Mohseni, 2018; Sklenarova et al., 2018), the role of gender has received only limited attention. This study builds on earlier work by placing greater emphasis on gender differences and by investigating the full spectrum of OSA engagement across a broader adult population, rather than focusing primarily on younger adults. To avoid one-sided results leaning overly toward risks or benefits, we gave our survey participants the opportunity to report equally about their positive and negative OSA experiences. In doing so, we aim to provide a more comprehensive understanding of OSA that can support education, counseling, and sexual health initiatives.

### Definition of Online Sexual Activities

The umbrella term OSA refers to Internet-based activities, content, and interactions that are sexual in nature (Shaughnessy et al., 2017). Typically, OSA include six main types of activities that are also common in offline contexts: (1) searching for sexual and reproductive health information (e.g., retrieving contraception information), (2) seeking sexual entertainment (e.g., getting sexually stimulating stories or videos), (3) initiating or maintaining sexual contact (e.g., finding new sexual partners via dating platforms/apps or sexting with established partners), (4) participating in communities for sexual minorities or specific sexual interests (e.g., lesbian, gay, or asexual online groups), (5) purchasing sex-related products (e.g., sex toys), and (6) selling or buying sexual services (e.g., commercial live webcam shows; Döring et al., 2017). Each type can be further differentiated; for example, sexual entertainment may vary by content format and mode of access (Döring et al., 2021). These categories reflect distinct ways of engaging in sexual activities with and through digital media—individually or with others, with or without sexual arousal—which the OSA framework captures through its action-oriented focus on how individuals actively shape their sexual experiences online (Barrada et al., 2019; Döring & Mohseni, 2018; Döring et al., 2021; Shaughnessy et al., 2011).

### Relevance of Online Sexual Activities for Sexual Health

Depending on biological, psychological, social, economic, and technological context factors, OSA can shape various aspects of individuals’ lives, including sexual knowledge and attitudes, identities, and behaviors (Barrada et al., 2019; Courtice et al., 2021; Coyne et al., 2022; Döring & Mohseni, 2018). Some outcomes are subjectively experienced and evaluated as positive or beneficial, while others are perceived as negative or risky (Castro-Calvo et al., 2018; Cooper et al., 2004; Naezer, 2018).

#### Benefits of Online Sexual Activities

Engaging in OSA may help individuals fulfill sexual needs, explore new experiences, improve pleasure and well-being, and expand opportunities for intimacy (Ballester-Arnal et al., 2025; Kohut et al., 2017). OSA can support partner search, relationship formation, and intimate communication, thereby reducing social isolation and enhancing social participation (Döring & Mohseni, 2018; Herbenick et al., 2020). OSA are also associated with greater sexual and relationship satisfaction, improved self-esteem and body image, and increased sexual confidence (Barrada et al., 2019; Coduto, 2024; Herbenick et al., 2020). In addition, digital media can facilitate the exploration of sexuality and identity, promote sexual and reproductive health knowledge, and offer access to information, support, and safe spaces—particularly valuable for sexual minorities who may lack such resources offline (Barrada et al., 2019; Coyne et al., 2022; Döring et al., 2017; Herbenick et al., 2020).

#### Risks of Online Sexual Activities

Despite their potential benefits, OSA can also be associated with a range of harmful outcomes, including harassment, victimization, stalking, sextortion, abuse of shared content, unwanted exposure to explicit material, involvement in unethical and illegal activities, and incidents of perceived infidelity (Coyne et al., 2022; Döring, 2009; Döring & Mohseni, 2018; Liu & Zheng, 2019; Sklenarova et al., 2018). Such experiences can lead to emotional distress, feelings of guilt or vulnerability, self-harm and harm of others, loss of control, mistrust, changes in behavior, anxiety, depression, and reduced relationship satisfaction (Cooper et al., 2004; Döring et al., 2017; Herbenick et al., 2020). In some cases, individuals may develop patterns of compulsive or dysfunctional use, which can be associated with clinically relevant symptoms and personality disorders (Chapman et al., 2025; Hermand et al., 2020; Vaillancourt-Morel et al., 2017).

### Prevalence of Online Sexual Activities

OSA are prevalent in large groups of Internet users in different countries (Ballester-Arnal et al., 2021; Döring et al., 2017; Sevcikova et al., 2023; Sklenarova et al., 2018). A 2015 survey in Germany indicated that up to 68% of adult Internet users had engaged in at least one type of OSA at least once in their lifetime—most commonly seeking sexual information, viewing pornography, or sexting (Döring & Mohseni, 2018). Gender is a key predictor of OSA engagement, with men of all age groups being more likely to engage in OSA than women (Cooper et al., 2004; Döring & Mohseni, 2018; Herbenick et al., 2020; Scandurra et al., 2022). Gender differences also appear in patterns of problematic and excessive use (Giordano & Cashwell, 2017), motives for engagement (Ballester-Arnal et al., 2023), and online experiences—both positive and negative (Döring et al., 2017). Women, for example, report more frequent pressure to sext and are more likely to receive unsolicited sexually explicit messages (Barrada et al., 2019; Döring et al., 2017; Mori et al., 2022). These patterns likely reflect a complex interplay of biological, psychological, and social factors including gender stereotypes and sexual double standards that influence how men and women express and experience sexuality, both offline and online (Nimbi et al., 2022; Santoniccolo et al., 2023).

However, much of the existing research focuses on only selected forms of OSA—such as sexting or pornography consumption—or on younger populations, providing only a fragmented view of OSA engagement and outcomes.

### The Current Study

Drawing on these considerations, as well as both the risk-oriented and benefit-oriented theoretical perspectives on OSA outcomes introduced above, we aimed to investigate four research questions (RQ): the 12-month prevalence of any type of engagement in OSA among adults in Germany (RQ1); the 12-month prevalence of the main types of OSA (RQ2); the extent to which engagement in the different types of OSA is experienced as positive or negative (RQ3); and the perceived overall personal outcomes of OSA, both positive and negative (RQ4). In addition, our specific focus was on how these aspects vary by gender.

## Method

To answer our study’s research questions, we conducted a survey of a national online sample of adults in Germany. The study follows an Open Science approach: We have made our instrument, non-identifiable data, and analysis script publicly available (https://osf.io/wf9c6).

### Participants

We surveyed participants aged 18–65 residing in Germany via an incentivized online panel managed by Bilendi, a global provider of data collection and technology solutions for market and social research. Participants voluntarily join this quality-controlled opt-in panel and are typically invited to take part in specific surveys in exchange for a small monetary reward (e.g., between 0.50 and 1.00 Euro per survey).

Bilendi operates under ISO 20252:2019 standards for market, opinion, and social research. Their data collection and processing fully comply with the EU General Data Protection Regulation and German national data protection law. They are members of various European professional associations for market research, and they implement rigorous quality controls at all panel lifecycle stages, including multi-source recruitment, double opt-in registration, internal source scoring, continuous behavioral monitoring, and regular panel refreshment, as explained on their website.^1^ All participants were registered members of the Bilendi online panel and provided informed consent before taking part in the study. Participation was voluntary. Respondents could pause the questionnaire, return to it later, or withdraw at any time.

Bilendi employed an uncrossed quota sampling procedure to approximate the Internet-using population in Germany aged 18 to 65 years, based on age, gender, education, and federal state. No weights or cluster-based sampling methods were applied. A total of 95,290 panelists were invited to participate in the survey, of whom 7028 (7.4%) opened the questionnaire and 2969 completed it during the two-week fieldwork period. As part of the quality control process, 137 responses were excluded due to indicators of low data quality, including unrealistically fast completion times and/or implausible or meaningless entries in open-ended fields. This data cleaning procedure did not distort the original quota distribution (see Table 1). The average age of participants was 43.8 years (*SD* = 13.4), with half self-identifying as women. Table 1 summarizes characteristics of the final study sample of 2832 participants.

**Table 1:** Sociodemographic characteristics of online survey participants in Germany (*N* = 2832), absolute and relative frequencies and comparison with target distribution

Characteristic	Participants		Target distribution	*ꭓ*^2^	*df*	*p*
	*n*	%	%			
Sex				1.57	1	.21
Female	1421	50	49			
Male	1411	50	51			
Age				4.58	4	.33
18–29	588	21	22			
30–39	548	19	20			
40–49	575	20	20			
50–59	736	26	25			
60–65	385	14	13			
Marital status				3.35	1	.06
Unmarried	1266	45	43			
Married	1566	55	57			
Education				0.72	2	.70
Low	725	26	26			
Moderate	984	35	34			
High	1123	40	40			
Federal state of Germany				1.26	15	1.00
Baden-Württemberg	374	13	13			
Bavaria	460	16	16			
Berlin	116	4	4			
Brandenburg	81	3	3			
Bremen	27	1	1			
Hamburg	57	2	2			
Hesse	195	7	7			
Mecklenburg-Western Pomerania	52	2	2			
Lower Saxony	284	10	10			
North Rhine-Westphalia	647	23	23			
Rhineland-Palatinate	141	5	5			
Saarland	26	1	1			
Saxony	141	5	5			
Saxony-Anhalt	86	3	3			
Schleswig–Holstein	87	3	3			
Thuringia	58	2	2			
Sexual identity				N/A^a^		
Heterosexual	2535	90				
Homosexual	111	4				
Bisexual	141	5				
Other	45	2				

The targeted distribution is based on the b4p 2021 structural analysis with Sinus Milieus (https://gik.media/best-4-planning/), a widely used quota plan in online survey research in Germany. The chi-square tests show whether the observed absolute frequencies deviate from the targeted b4p quotas. Percentage values are rounded

^a^No quota plan was available for sexual identity

### Procedure

We administered the questionnaire online using EFS survey from QuestBack Unipark.^2^ Eligible participants received an invitation link via email from the panel provider. Data collection was conducted within a focused two-week timeframe, from November 17 to December 1, 2022, for logistic reasons and to avoid the upcoming holiday season. The average completion time was 8 min, with a median of 6 min.

### Measures

We developed the questionnaire primarily based on previous studies on the prevalence of different types of OSA (Döring & Mohseni, 2018; Döring et al., 2017). It included items on participants’ sociodemographic characteristics, their engagement in the main types of OSA, their positive or negative experiences with the main types of OSA, as well as their perceived positive and negative outcome of sexuality in digital media, understood in terms of OSA in general (for the questionnaire, see https://osf.io/wf9c6).

Sociodemographic variables such as participants’ gender, age, marital status, education, and current federal state were collected as quota variables via Bilendi’s servers. Gender was recorded as a binary variable due to limitations in the panel provider’s system at the time, requiring participants to self-identify as either male or female as no quota was available for other gender identities. Additionally, we asked participants to report their sexual identity.

To assess the prevalence of OSA, participants were asked to report their engagement in the following six types of OSA during the past 12 months: (1) sexual information, (2) sexual entertainment, (3a) sexual contact—searching for sexual contact online, (3b) sexual contact—sexting, (4) sexual communities, (5) sex products, and (6) sex work. We explicitly distinguished between two types of sexual contact—searching for new partners online and sexting with established partners—based on their separate classification in earlier research (Döring & Mohseni, 2018; Döring et al., 2017). The overall prevalence of OSA was calculated by identifying participants who reported engaging in at least one of these activities during the past 12 months. We did not use one of the three earlier OSA measures (as summarized in Eleuteri et al., 2014) because they do not capture the broad spectrum of OSA, but instead focus primarily on only three subdimensions: (1) sexual information seeking or non-arousal OSA, (2) sexually explicit material or solitary arousal OSA, and (3) sexual contacts/partners or partnered arousal OSA (Goodson et al., 2000; Shaughnessy et al., 2011; Velezmoro et al., 2012).

Participants who had engaged in OSA rated their overall experience with each type they had used by answering the question “What is your overall experience with [specific OSA]?”, on a 5-point rating scale ranging from 1 (very bad) to 5 (very good; a similar scale for evaluating subjective experiences with OSA was used in other surveys such as in Sklenarova et al., 2018).

In addition, participants were asked to evaluate how their OSA engagement across all types of OSA had affected them personally. Drawing on prior work (Döring & Mohseni, 2018; Döring et al., 2017), we measured perceived positive and negative OSA outcomes as two independent dimensions. Each was assessed separately using a 7-point rating scale in response to the question: “To what extent has sexuality in digital media had a positive [negative] outcome on you personally?”, with response options on a 7-point rating scale ranging from 1 (not at all) to 7 (to a great extent).

### Statistical Analysis

We analyzed the data using R version 4.5.0. OSA prevalence was estimated with 99% confidence intervals based on multinomial methods implemented in the *DescTools* package. Gender differences were examined using chi-square tests and independent samples t-tests using *gmodels* and *rstatix* packages, respectively. To account for the large sample size, we applied a stricter significance threshold of 1% to reduce the risk of Type I errors. While multiple comparisons were conducted across gender and OSA types, each test examined a conceptually distinct behavior rather than repeated measures of the same construct. Therefore, no additional correction (e.g., Bonferroni) was applied. For the complete R script see https://osf.io/wf9c6.

## Results

We present the survey results separately for each of the study’s four research questions.

### Prevalence of Online Sexual Activities Overall

Overall, 1755 respondents (62%) reported engaging in at least one type of OSA within the past 12 months (see Table 2). The overall 12-month prevalence was significantly higher among men (78%) than women (47%), *ꭓ*^2^(1) = 286.39, *p* < .001, *V* = .32, indicating a medium effect size (RQ1).

**Table 2:** Engagement in different types of online sexual activities among online survey participants in Germany (*n* = 2832), absolute and relative 12-month prevalences

Variables	Total (*N* = 2832)		Women (*n* = 1421)		Men (*n* = 1411)		ꭓ^2^(1)	*p*	*V*
	*n*	% CI	*n*	% CI	*n*	% CI			
OSA	1755	62.0 [59.7, 64.3]	662	46.6 [43.2, 50.1]	1093	77.5 [74.9, 80.0]	286.39	< .001	.32
Sexual information	1308	46.2 [43.8, 48.6]	486	34.2 [31.1, 37.3]	822	58.3 [54.9, 61.7]	164.83	< .001	.24
Sexual entertainment	1457	51.4 [49.0, 53.9]	453	31.9 [28.9, 34.5]	1004	71.2 [68.3, 74.1]	437.23	< .001	.39
Sexual contact search	380	13.4 [12.0, 14.8]	107	7.5 [6.1, 9.1]	273	19.3 [17.0, 21.8]	85.11	< .001	.17
Sexting	450	15.9 [14.4, 17.4]	188	13.2 [11.3, 15.2]	262	18.6 [16.3, 21.0]	15.10	< .001	.07
Sexual communities	139	4.9 [4.1, 5.8]	30	2.1 [1.4, 2.9]	109	7.7 [6.2, 9.2]	47.81	< .001	.13
Sex products	401	14.2 [12.7, 15.6]	179	12.6 [10.7, 14.5]	222	15.7 [13.6, 17.9]	5.73	.02	.04
Sex work	76	2.7 [2.1, 3.3]	14	1.0 [0.5, 1.5]	62	4.4 [3.3, 5.6]	31.50	< .001	.10

OSA = Online Sexual Activities. CI = 99% Confidence Interval. Statistical significance threshold was set to 1% due to a large sample size

### Prevalence of Different Online Sexual Activities Types

The most frequently reported OSA type was accessing sexually stimulating material (51%), followed by seeking sexuality-related information (46%). These two types of OSA were also the most commonly reported within each gender group. As shown in Table 2, there were statistically significant gender differences of small to medium effect size in engagement across most OSA types, with men consistently reporting more frequent use than women—except in the case of purchasing sex-related products which did not demonstrate statistically significant gender differences (RQ2).

### Experiences with Different Online Sexual Activities Types

Purchasing sex products online was experienced as the most positive type of OSA, while searching for sexual contacts online received the most negative ratings (see Table 3). Between men and women, a statistically significant difference emerged only in experiences with sexual information and sexual entertainment, with men rating their experiences more positively than women (see Tables 3, 4; RQ3).

**Table 3:** Subjective experiences with online sexual activities among online survey participants in Germany (*N* = 2832)

OSA type	% total sample (*N* = 2832)	*n*	*M* (*SD*)	Subjective experience	
				% positive	% negative
All participants with 12-month prevalence (*n* = 1755)					
Sexual information	46	1308	3.63 (0.77)	57	4
Sexual entertainment	51	1457	3.68 (0.82)	59	6
Sexual contact search	13	380	3.08 (1.10)	33	29
Sexting	16	450	3.85 (0.83)	70	5
Sexual communities	5	139	3.55 (0.98)	55	12
Sex products	14	401	4.13 (0.76)	83	3
Sex work	3	76	3.87 (0.90)	70	7
Women with 12-month prevalence (*n* = 662)					
Sexual information	17	486	3.53 (0.71)	51	4
Sexual entertainment	16	453	3.48 (0.81)	47	9
Sexual contact search	4	107	3.06 (1.05)	33	30
Sexting	7	188	3.78 (0.80)	70	5
Sexual communities	1	30	3.50 (1.14)	47	13
Sex products	6	179	4.13 (0.80)	82	3
Sex work	< 1	14	3.43 (0.85)	50	14
Men with 12-month prevalence (*n* = 1093)					
Sexual information	29	822	3.69 (0.80)	60	4
Sexual entertainment	35	1004	3.77 (0.80)	64	4
Sexual contact search	10	273	3.08 (1.12)	33	29
Sexting	9	262	3.90 (0.85)	71	5
Sexual communities	4	109	3.57 (0.94)	58	12
Sex products	8	222	4.12 (0.74)	84	3
Sex work	2	62	3.97 (0.89)	74	5

OSA = Online Sexual Activities. Subjective experiences were measured on a 5-point rating scale from 1 (very bad) to 5 (very good). Positive experiences included values 4 (good) and 5 (very good). Negative experiences included values 1 (very bad) and 2 (bad). Percentages reflect the proportion of positive and negative responses among all reported experiences for each OSA type

**Table 4:** Differences between surveyed women OSA practitioners (*n* = 662) and men OSA practitioners (*n* = 1093) in their subjective experiences with different types of online sexual activities

OSA type	*t*	*df*	*p*	*diff*	CI	Cohen’s *d*
Sexual information	3.55	1306	< .001	0.16	[0.04, 0.27]	0.21
Sexual entertainment	6.41	1455	< .001	0.29	[0.17, 0.41]	0.36
Sexual contact search	0.17	378	.87	0.02	[− 0.30, 0.35]	0.02
Sexting	1.50	448	.13	0.12	[− 0.09, 0.32]	0.14
Sexual communities	0.34	137	.73	0.07	[− 0.46, 0.60]	0.07
Sex products	− 0.16	399	.87	− 0.01	[− 0.21, 0.19]	− 0.02
Sex work	2.07	74	.04	0.54	[− 0.15, 1.23]	0.61

OSA = Online Sexual Activities. Subjective experiences were measured on a 5-point rating scale from 1 (very bad) to 5 (very good). CI = 99% confidence interval. diff = mean value difference: *M*(men) − *M*(women). Statistical significance threshold was set to 1% due to a large sample size

### Subjective Outcomes of Online Sexual Activities Overall

Among participants who had engaged in OSA in the past 12 months, approximately 42% reported perceiving no negative outcomes on their personal life, while 2% reported a strong negative outcome. In contrast, 17% indicated no perceived positive outcomes, and 6% reported a strong positive outcome. Participants perceived the positive outcomes of OSA more strongly than the negative ones, both overall and within each gender group, indicating large effect sizes (see Table 5; RQ4). A total of 244 participants (14% of all OSA users) reported experiencing neither positive nor negative outcomes from OSA, including 17% of women and 12% of men.

**Table 5:** Differences within surveyed women (*n* = 662) and men (*n* = 1093) in their perceived positive and negative outcomes of online sexual activities in general

	*n*	Perceived negative outcome			Perceived positive outcome			*t*	*df*	*p*	Cohen’s *d*
		*M (SD)*	% no negative outcome	% strong negative outcome	*M (SD)*	% no positive outcome	% strong positive outcome				
Total	1755	2.46 (1.62)	42	2	3.89 (1.77)	17	6	27.63	1754	< .001	0.84
Women	662	2.39 (1.54)	42	1	3.67 (1.67)	20	3	16.36	661	< .001	0.78
Men	1093	2.50 (1.66)	41	2	4.03 (1.79)	16	7	22.33	1092	< .001	0.89

Perceived outcome was measured on two 7-point rating scales. “No outcome” considers the value 1 (not at all); “Strong outcome” considers the value 7 (to a great extent). Statistical significance threshold was set to 1% due to a large sample size

## Discussion

Our results confirm and expand earlier survey research describing OSA as a relatively common behavior in many countries and populations (Kim & Berdychevsky, 2025; Scandurra et al., 2022), including adults in Germany (Döring & Mohseni, 2018; Sklenarova et al., 2018). While a 2015 national sample of adults in Germany reported a 68% OSA lifetime prevalence (Döring & Mohseni, 2018), seven years later we found a 62% 12-month prevalence, underscoring the ongoing normalization of OSA (see Table 2). Overall OSA engagement was more frequently reported by men (78%) than women (47%), indicating a medium effect size (see Table 2). More frequent overall (RQ1) and specific (RQ2) OSA engagement among men is consistent with earlier studies, particularly regarding their more frequent use of sexually explicit online material, reflecting well-documented gender differences in masturbation and masturbatory pornography use (Ballester-Arnal et al., 2021; Döring & Mohseni, 2018; Döring et al., 2017; Scandurra et al., 2022).

Across the spectrum of different types of OSA, both men and women consistently reported much stronger positive than negative experiences (see Tables 3, 4; RQ3)—a novel finding, as earlier studies did not cover all main OSA types and practitioners’ experiences with them. Purchasing sex products online stood out as the type of OSA associated with the most positive (83%) and least negative (3%) experiences across genders. This aligns with research suggesting that online shops have made access to sex products —such as sex toys, lingerie, or other sexual merchandise—easier, more effective, and largely free from shame, guilt, or insecurity: Customers can make informed decisions in the privacy of their own homes based on product photos, videos, and reviews (Döring & Poeschl, 2020). In contrast, searching for sexual partners online—for example through dating platforms or apps—was reported as the least positive (33%) and most negative (29%) experience across genders (see Table 3). Some respondents provided explanations for their negative evaluations in an open-ended response field: Several men, for example, expressed frustration about online dates who never showed up or did not resemble their online profiles, while some women reported the same issues and also mentioned experiences of violence, such as receiving unwanted sexual images (so-called “dick pics”) or encountering harassment from their online dates. These anecdotes are in line with previous research that points to different and gendered challenges and risks of online dating (Döring, 2009). However, these results should be interpreted with caution, as the questionnaire item wording combined the search for romantic and sexual partners under a single response option. It is possible that negative experiences reflect, at least in part, the frustration of unmet romantic expectations rather than dissatisfaction with casual sexual encounters per se. Earlier research suggests that online partner seeking can evoke different emotional reactions depending on users’ intentions and relational goals (Ward, 2017). Differentiating between romantic and sexual motivations in future surveys might reveal more nuanced patterns of experience and help clarify which aspects of online partner search are associated with negative feelings.

Finally, the surveyed adults in Germany reported moderately positive subjective outcomes of their OSA, with a mean value of 3.89 on the 7-point rating scale. In contrast, negative outcomes were rated much lower, with a mean of 2.46 (see Table 5; RQ4). These results are roughly consistent with those of a 2015 OSA survey conducted among adults in Germany that found a positive subjective outcome of 2.98 for OSA and of 3.64 for sexting on the same 7-point scale, compared to a negative outcome of 2.10 for OSA and 2.20 for sexting (Döring & Mohseni, 2018). Against the backdrop of public and academic discourses that often emphasize the challenges and risks of OSA, our results demonstrate that OSA-involved women and men consistently report experiencing significantly more advantages than disadvantages.

### Limitations and Research Directions

The present study benefits from surveying a large national sample of adults in Germany (aged 18–65). In contrast to previous studies, which often relied on small convenience samples and focused on selected types of OSA or primarily negative aspects, this survey covered the full spectrum of main OSA types and allowed respondents to report both positive and negative experiences and outcomes in a balanced manner.

However, several limitations need to be considered. While our sample mirrors the adult online population in Germany in key sociodemographic variables, a quota sample from an online panel is still more biased than a random sample. In particular, the study invitation might have attracted people engaged in OSA to a higher degree, leading to an overestimation of OSA involvement. Furthermore, our sample does not adequately represent minority gender identities. Future survey research should focus on the inclusion of more gender diversity, particularly among non-binary, transgender, and intersex populations, who often experience higher rates of prejudice and discrimination related to their sexual activities—making online spaces potentially especially valuable for them (Coyne et al., 2022). However, the online empowerment and identity validation benefits of sexual and gender minorities through OSA types such as online community engagement may also entail risks of disempowerment (e.g., due to online hate speech), which warrant further exploration (Döring et al., 2022).

Our survey methodology required the use of standardized questions to include a large sample, whereas a qualitative oral interview approach with open questions and a smaller sample can produce data that reflect more detail of individual OSA involvement and its context. However, both quantitative and qualitative self-report data are subject to various biases, including recall errors, social desirability, misinterpretation of questions, and differences in self-awareness or willingness to disclose sensitive information. Future studies could complement subjective data with objective data on both OSA engagement (such as log-file recordings on digital devices) and OSA outcomes (such as psychometric tests of sexual health).

Another important limitation concerns the level of specificity in how OSA outcomes were assessed. While our survey allowed participants to indicate general positive and negative effects, it did not differentiate between particular domains such as sexual pleasure, emotional well-being, or relational dynamics. This limits the depth of insight into how different OSA types may affect specific areas of life. Future research should explore these differentiated outcome dimensions, which may vary not only by OSA type but also by users’ intentions, relational contexts, and gender.

While OSA is a well-established umbrella term, and the main types of OSA have remained stable and relevant over the past decades, future studies should accommodate technological change. The integration of artificial intelligence (AI) in OSA, for example, should be acknowledged by measuring AI-enabled OSA subtypes such as engagement in sexual interactions with AI agents (Döring et al., 2025; Hanson & Bolthouse, 2024).

### Conclusion

To support sexual health and well-being in the digital age, professionals—including sex educators, counselors, sexual health experts, and digital platform providers—are encouraged to adopt a nuanced, evidence-informed approach to OSA. Rather than framing OSA primarily as problem behaviors, these practices can be acknowledged for their potential to foster sexual expression and subjective well-being. The high and growing prevalence of OSA, along with predominantly positive user experiences, underscores the importance of destigmatizing digital sexual practices and meaningfully integrating them into sexual education, counseling, and healthcare. Practitioners can help individuals navigate both offline and online sexuality in a supportive, nonjudgmental way, while also addressing risks such as harassment and gendered harms linked, for example, to online partner search. This calls for user-centered safety features on digital platforms, critical media literacy in education, and gender-sensitive counseling strategies.

## Data Availability

The study instrument, data, and analysis script are publicly available at https://osf.io/wf9c6.

## Footnotes

1 https://www.bilendi.com/, last accessed: June 16, 2025.

2 https://www.unipark.com, last accessed: June 16, 2025.

## References

[CR1] Ballester-Arnal, R., Castro-Calvo, J., García-Barba, M., Ruiz-Palomino, E., & Gil-Llario, M. D. (2021). Problematic and non-problematic engagement in online sexual activities across the lifespan. *Computers in Human Behavior,**120*, Article 106774. 10.1016/j.chb.2021.106774

[CR2] Ballester-Arnal, R., García-Barba, M., Castro-Calvo, J., Giménez-García, C., & Gil-Llario, M. D. (2023). Pornography consumption in people of different age groups: An analysis based on gender, contents, and consequences. *Sexuality Research & Social Policy,**20*(2), 766–779. 10.1007/s13178-022-00720-z

[CR3] Ballester-Arnal, R., García-Barba, M., Elipe-Miravet, M., Castro-Calvo, J., & Gil-Llario, M. D. (2025). Changes in online sexual activities during the lockdown caused by COVID-19 in Spain: “INSIDE” project. *Sexuality Research & Social Policy,**22*(2), 1083–1101. 10.1007/s13178-024-00987-4 10.1007/s13178-020-00506-1 PMC7666970 33224310

[CR4] Barrada, J. R., Ruiz-Gómez, P., Correa, A. B., & Castro, Á. (2019). Not all online sexual activities are the same. *Frontiers in Psychology,**10*, Article 339. 10.3389/fpsyg.2019.00339 30863340 10.3389/fpsyg.2019.00339 PMC6399151

[CR5] Castro-Calvo, J., Giménez-García, C., Gil-Llario, M. D., & Ballester-Arnal, R. (2018). Motives to engage in online sexual activities and their links to excessive and problematic use: A systematic review. *Current Addiction Reports,**5*(4), 491–510. 10.1007/s40429-018-0230-y

[CR6] Chapman, M., Dammeyer, J., Strizzi, J. M., & Hald, G. M. (2025). Using pornography, paying for sex, and violence: A Danish national survey study. *Journal of Sex Research, 62*, 1037–1048. 10.1080/00224499.2023.2280965 38063496 10.1080/00224499.2023.2280965

[CR7] Coduto, K. D. (2024). Channel affordances for sexting: Social presence relates to improved self-esteem, sexual gratification, and sexting certainty. *Sexuality & Culture,**28*(1), 228–242. 10.1007/s12119-023-10112-z

[CR8] Cooper, A. L., Delmonico, D. L., Griffin-Shelley, E., & Mathy, R. M. (2004). Online sexual activity: An examination of potentially problematic behaviors. *Sexual Addiction & Compulsivity,**11*(3), 129–143. 10.1080/10720160490882642

[CR9] Courtice, E. L., Shaughnessy, K., Blom, K., Asrat, Y., Daneback, K., Döring, N., Grov, C., & Byers, E. S. (2021). Young adults’ qualitative self-reports of their outcomes of online sexual activities. *European Journal of Investigation in Health, Psychology and Education,**11*(2), 303–320. 10.3390/ejihpe11020023 34708815 10.3390/ejihpe11020023 PMC8314359

[CR10] Coyne, C. A., Wongsomboon, V., Korpak, A. K., & Macapagal, K. (2022). “we have to figure it out ourselves”: Transfeminine adolescents’ online sexual experiences and recommendations for supporting their sexual health and wellbeing. *Frontiers in Reproductive Health,**4*, 1034747. 10.3389/frph.2022.1034747 36726593 10.3389/frph.2022.1034747 PMC9884802

[CR11] Desbuleux, J. C., Desbuleux, J. F. M., & Fuss, J. (2025). Prevalence of first- and second-wave digisexualities in Germany and their relation to compulsive sexual behavior: Findings from a national online survey. *Journal of Behavioral Addictions,**14*(2), 1040–1050. 10.1556/2006.2025.00048 40459929 10.1556/2006.2025.00048 PMC12231451

[CR12] Döring, N. (2000). Feminist views of cybersex: Victimization, liberation, and empowerment. *Cyberpsychology & Behavior,**3*(5), 863–884. 10.1089/10949310050191845

[CR13] Döring, N. (2009). The internet’s impact on sexuality: A critical review of 15 years of research. *Computers in Human Behavior,**25*(5), 1089–1101. 10.1016/j.chb.2009.04.003

[CR14] Döring, N. (2012). Internet sexuality. In Z. Yan (Ed.). *Encyclopedia of cyber behavior* (pp. 807–827). IGI Global Scientific Publishing. 10.4018/978-1-4666-0315-8.ch067

[CR15] Döring, N., Bhana, D., & Albury, K. (2022). Digital sexual identities: Between empowerment and disempowerment. *Current Opinion in Psychology,**48*, Article 101466. 10.1016/j.copsyc.2022.101466 36242854 10.1016/j.copsyc.2022.101466

[CR16] Döring, N., Daneback, K., Shaughnessy, K., Grov, C., & Byers, E. S. (2017). Online sexual activity experiences among college students: A four-country comparison. *Archives of Sexual Behavior,**46*(6), 1641–1652. 10.1007/s10508-015-0656-4 26659580 10.1007/s10508-015-0656-4

[CR17] Döring, N., Krämer, N., Mikhailova, V., Brand, M., Krüger, T. H. C., & Vowe, G. (2021). Sexual interaction in digital contexts and its implications for sexual health: A conceptual analysis. *Frontiers in Psychology,**12*, Article 769732. 10.3389/fpsyg.2021.769732 34916999 10.3389/fpsyg.2021.769732 PMC8669394

[CR18] Döring, N., Le, T. D., Vowels, L. M., Vowels, M. J., & Marcantonio, T. L. (2025). The impact of artificial intelligence on human sexuality: A five-year literature review 2020–2024. *Current Sexual Health Reports,**17*(1), 4. 10.1007/s11930-024-00397-y

[CR19] Döring, N., & Mohseni, M. R. (2018). Are online sexual activities and sexting good for adults’ sexual well-being? Results from a national online survey. *International Journal of Sexual Health,**30*(3), 250–263. 10.1080/19317611.2018.1491921

[CR20] Döring, N., & Poeschl, S. (2020). Experiences with diverse sex toys among German heterosexual adults: Findings from a national online survey. *Journal of Sex Research,**57*(7), 885–896. 10.1080/00224499.2019.1578329 30806076 10.1080/00224499.2019.1578329

[CR21] Eleuteri, S., Tripodi, F., Petruccelli, I., Rossi, R., & Simonelli, C. (2014). Questionnaires and scales for the evaluation of the online sexual activities: A review of 20 years of research. *Cyberpsychology: Journal of Psychosocial Research on Cyberspace,**8*(1), Article 2. 10.5817/CP2014-1-2

[CR22] Giordano, A. L., & Cashwell, C. S. (2017). Cybersex addiction among college students: A prevalence study. *Sexual Addiction & Compulsivity,**24*(1–2), 47–57. 10.1080/10720162.2017.1287612

[CR23] Goodson, P., McCormick, D., & Evans, A. (2000). Sex and the internet: A survey instrument to assess college students’ behavior and attitudes. *Cyberpsychology & Behavior,**3*(2), 129–149. 10.1089/109493100315987

[CR24] Griffin-Shelley, E. (2003). The internet and sexuality: A literature review–1983-2002. *Sexual and Relationship Therapy,**18*(3), 355–370. 10.1080/1468199031000153955

[CR25] Hanson, K. R., & Bolthouse, H. (2024). Replika removing erotic role-play is like Grand Theft Auto removing guns or cars”: Reddit discourse on artificial intelligence chatbots and sexual technologies. *Socius: Sociological Research for a Dynamic World, 10*. 10.1177/23780231241259627

[CR26] Herbenick, D., Fu, T.-C., Wright, P., Paul, B., Gradus, R., Bauer, J., & Jones, R. (2020). Diverse sexual behaviors and pornography use: Findings from a nationally representative probability survey of Americans aged 18 to 60 years. *Journal of Sexual Medicine,**17*(4), 623–633. 10.1016/j.jsxm.2020.01.013 32081698 10.1016/j.jsxm.2020.01.013

[CR27] Hermand, M., Benyamina, A., Donnadieu-Rigole, H., Petillion, A., Amirouche, A., Roméo, B., & Karila, L. (2020). Addictive use of online sexual activities and its comorbidities: A systematic review. *Current Addiction Reports,**7*(2), 194–209. 10.1007/s40429-020-00301-3

[CR28] Hertlein, K. M., & Cravens, J. D. (2014). Assessment and treatment of internet sexuality issues. *Current Sexual Health Reports,**6*(1), 56–63. 10.1007/s11930-013-0011-5

[CR29] Kim, S., & Berdychevsky, L. (2025). Cybersex as leisure: Socio-demographic and socio-psychological profiling of the (non)participants in online sexual activities. *Leisure Sciences*. 10.1080/01490400.2025.2503974

[CR30] Kohut, T., Fisher, W. A., & Campbell, L. (2017). Perceived effects of pornography on the couple relationship: Initial findings of open-ended, participant-informed, “bottom-up” research. *Archives of Sexual Behavior,**46*(2), 585–602. 10.1007/s10508-016-0783-6 27393037 10.1007/s10508-016-0783-6

[CR31] Liu, Y., & Zheng, L. (2019). Influences of sociosexuality and commitment on online sexual activities: The mediating effect of perceptions of infidelity. *Journal of Sex & Marital Therapy,**45*(5), 395–405. 10.1080/0092623X.2018.1549632 30640583 10.1080/0092623X.2018.1549632

[CR32] Lunde, C., & Joleby, M. (2021). Young people’s experiences of sexting and online sexual victimization. In Y. Odenbring & T. Johansson (Eds.). *Violence, victimisation and young people: Education and safe learning environments* (Vol. 4, pp. 95–112). Springer. 10.1007/978-3-030-75319-1_7

[CR33] Machimbarrena, J. M., Calvete, E., Fernández-González, L., Álvarez-Bardón, A., Álvarez-Fernández, L., & González-Cabrera, J. (2018). Internet risks: An overview of victimization in cyberbullying, cyber dating abuse, sexting, online grooming and problematic internet use. *International Journal of Environmental Research and Public Health, 15*(11), 3471. 10.3390/ijerph15112471 10.3390/ijerph15112471 PMC6267617 30400659

[CR34] McArthur, N., & Twist, M. L. C. (2017). The rise of digisexuality: Therapeutic challenges and possibilities. *Sexual and Relationship Therapy,**32*(3–4), 334–344. 10.1080/14681994.2017.1397950

[CR35] Mori, C., Park, J., Temple, J. R., & Madigan, S. (2022). Are youth sexting rates still on the rise? A meta-analytic update. *Journal of Adolescent Health*, *70*(4), 531–539. 10.1016/j.jadohealth.2021.10.026 10.1016/j.jadohealth.2021.10.026 34916123

[CR36] Naezer, M. (2018). From risky behaviour to sexy adventures: Reconceptualising young people’s online sexual activities. *Culture, Health & Sexuality,**20*(6), 715–729. 10.1080/13691058.2017.1372632 10.1080/13691058.2017.1372632 28922092

[CR37] Nimbi, F. M., Galizia, R., Rossi, R., Limoncin, E., Ciocca, G., Fontanesi, L., Jannini, E. A., Simonelli, C., & Tambelli, R. (2022). The biopsychosocial model and the sex-positive approach: An integrative perspective for sexology and general health care. *Sexuality Research & Social Policy,**19*(3), 894–908. 10.1007/s13178-021-00647-x

[CR38] Riva, G., Baños, R. M., Botella, C., Wiederhold, B. K., & Gaggioli, A. (2012). Positive technology: Using interactive technologies to promote positive functioning. *Cyberpsychology, Behavior, and Social Networking,**15*(2), 69–77. 10.1089/cyber.2011.0139 22149077 10.1089/cyber.2011.0139

[CR39] Santoniccolo, F., Trombetta, T., Paradiso, M. N., & Rollè, L. (2023). Gender and media representations: A review of the literature on gender stereotypes, objectification and sexualization. *International Journal of Environmental Research and Public Health, 20*(10), 5770. 10.3390/ijerph20105770 37239498 10.3390/ijerph20105770 PMC10218532

[CR40] Scandurra, C., Mezza, F., Esposito, C., Vitelli, R., Maldonato, N. M., Bochicchio, V., Chiodi, A., Giami, A., Valerio, P., & Amodeo, A. L. (2022). Online sexual activities in Italian older adults: The role of gender, sexual orientation, and permissiveness. *Sexuality Research & Social Policy,**19*(1), 248–263. 10.1007/s13178-021-00538-1

[CR41] Sevcikova, A., Gocieková, V., Stašek, A., Gottfried, J., & Daneback, K. (2023). Online pornography use and sexual satisfaction in association with relationship satisfaction among middle-aged and older people. *Cyberpsychology: Journal of Psychosocial Research on Cyberspace*. 10.5817/CP2023-4-6

[CR42] Shaughnessy, K., Byers, E. S., & Walsh, L. (2011). Online sexual activity experience of heterosexual students: Gender similarities and differences. *Archives of Sexual Behavior,**40*(2), 419–427. 10.1007/s10508-010-9629-9 20467798 10.1007/s10508-010-9629-9

[CR43] Shaughnessy, K., Fudge, M., & Byers, E. S. (2017). An exploration of prevalence, variety, and frequency data to quantify online sexual activity experience. *Canadian Journal of Human Sexuality,**26*(1), 60–75. 10.3138/cjhs.261-A4

[CR44] Sklenarova, H., Schulz, A., Schuhmann, P., Osterheider, M., & Neutze, J. (2018). Online sexual solicitation by adults and peers - Results from a population based German sample. *Child Abuse & Neglect,**76*, 225–236. 10.1016/j.chiabu.2017.11.005 29149683 10.1016/j.chiabu.2017.11.005

[CR45] Vaillancourt-Morel, M.-P., Blais-Lecours, S., Labadie, C., Bergeron, S., Sabourin, S., & Godbout, N. (2017). Profiles of cyberpornography use and sexual well-being in adults. *Journal of Sexual Medicine,**14*(1), 78–85. 10.1016/j.jsxm.2016.10.016 28011208 10.1016/j.jsxm.2016.10.016

[CR46] Velezmoro, R., Negy, C., & Livia, J. (2012). Online sexual activity: Cross-national comparison between United States and Peruvian college students. *Archives of Sexual Behavior,**41*(4), 1015–1025. 10.1007/s10508-011-9862-x 22083655 10.1007/s10508-011-9862-x

[CR47] Ward, J. (2017). What are you doing on Tinder? Impression management on a matchmaking mobile app. *Information, Communication & Society,**20*(11), 1644–1659. 10.1080/1369118X.2016.1252412

[CR48] Williams, D. J., Thomas, J., Prior, E., & Walters, W. (2015). Introducing a multidisciplinary framework of positive sexuality. *Journal of Positive Sexuality,**1*(1), 6–11. 10.51681/1.112

